# IL-33 in Rheumatic Diseases

**DOI:** 10.3389/fmed.2021.739489

**Published:** 2021-09-13

**Authors:** Yuanji Dong, Jixin Zhong, Lingli Dong

**Affiliations:** Department of Rheumatology and Immunology, Tongji Hospital, Tongji Medical College, Huazhong University of Science and Technology, Wuhan, China

**Keywords:** IL-33, alarmin, ST2, autoimmune, rheumatic disease

## Abstract

Interleukin-33 (IL-33) is a nuclear factor mainly expressed in barrier epithelium, endothelial cells, and fibroblast reticular cells. Some inflammatory cells also express IL-33 under certain conditions. The important role of IL-33 in allergic reactions, helminth infection, cancer, tissue fibrosis, chronic inflammation, organ transplantation, and rheumatic immune diseases has been extensively studied in recent years. IL-33 primarily activates various circulating and tissue-resident immune cells, including mast cell, group 2 innate lymphoid cell (ILC2), regulatory T cell (Treg), T helper 2 cell (Th2), natural killer cell (NK cell), and macrophage. Therefore, IL-33 plays an immunomodulatory role and shows pleiotropic activity in different immune microenvironments. The IL-33/serum stimulation-2 (ST2) axis has been shown to have a detrimental effect on rheumatoid arthritis, systemic lupus erythematosus, and other rheumatic diseases. Interestingly, IL-33 also plays a protective role in the repair of barrier epithelium and the activation of Tregs. Therefore, the role of IL-33/ST2 depends on the underlying pathological conditions in rheumatic diseases. This review focuses on the dual role of the IL-33/ST2 axis in rheumatic diseases.

## Introduction

Interleukin-33 (IL-33), a member of the IL-1 family, was first discovered in human tissues in 2003 and was originally defined as a nuclear factor of high endothelial venules (NF-HEV) (1). In 2005, Schmitz et al. reported that the C-terminal (amino acids from 112 to 270) of NF-HEV exhibited an IL-1-like three-dimensional folding and induced a type 2 immune response through binding to its receptor serum stimulation-2 (ST2) (2). In 2006, the identity between IL-33 and NF-HEV and its role as a chromatin-related nuclear factor was further confirmed (3). IL-33 is produced by various cell types such as endothelial cells, epithelial cells, macrophages, fibroblasts, adipose progenitor cells, and dendritic cells. Under conditions of cell damage, necrosis, necroptosis, stress, and virus infection, it is released as a pro-inflammatory factor and activates different types of immune cells (4–7). The role of IL-33 in type 2 immune diseases has been extensively studied in allergic reactions, asthma, and parasitic infections (6). However, it is well-known that HEV is involved in the activation and mobilization of lymphocytes, indicating that IL-33 may also be involved in chronic inflammation (8, 9). Rheumatic diseases are chronic inflammatory disorders in which the immune system attacks itself and organs of the body. As an incurable condition so far, it brings a heavy burden to individuals and society (10). A growing number of studies have demonstrated a critical role of the IL-33/ST2 axis in rheumatic diseases, including systemic lupus erythematosus (SLE), rheumatoid arthritis (RA), primary Sjögren's syndrome (pSS), systemic sclerosis (SSc), psoriatic arthritis (PsA), gout, IgG4-related diseases, and ankylosing spondylitis (AS), indicating a promising potential for IL-33/ST2-targeting therapy in rheumatic disease (10).

## Biology of IL-33

### The Distribution and Function of IL-33

Unlike some cytokines, which have classical secretion patterns, IL-33 is normally localized in the nucleus (11, 12). Although the localization of IL-33 in the cytoplasm has been reported in the literature, the results were not obtained under normal conditions. This ectopic expression was observed in murine cell line NIH3T3 that expressed tetracysteine-labeled human IL-33 by genetic engineering. Because the cysteine residues can change the folding of IL-33 and the fluorescence staining has not been tested by knockout, the results of cytoplasmic localization need to be treated with caution (13). The N-terminal domain of IL-33 shows evolutionary conservation and is closely related to the nuclear location of IL-33. The N-terminal domain of IL-33 was initially thought to contain homologous domain-like structures bound to deoxyribonucleic acid (DNA), but this has not been confirmed. In fact, IL-33 binds to DNA via protein–protein interactions. Through the tight hairpin structure of the chromatin-binding protein, it is combined with the acid pocket formed by histone 2A (H2A) and histone 2B (H2B) (1, 14). Although IL-33 is located in the nucleus, it does not appear to regulate the expression of genes. The nuclear localization of IL-33 seems to regulate the activity of IL-33 as a cytokine (15, 16). IL-33 is released outside of the cell and has a variety of immunological effects. Initially, researchers believed that the full-length IL-33 should be processed to be biologically active, and in the next few years, it was considered to be activated by caspase-1 and inflammasome, similar to IL-1β and IL-18. However, in 2009, Girard et al. reported that full-length IL-33 could interact with ST2 and activate nuclear factor-kappa B (NF-κB) activity to induce cytokine production (17, 18). Meanwhile, further studies also found the inflammatory protease hydrolysis site of IL-33. An 18- to 21-kilodalton (kDa) mature form can be produced when IL-33 is cleaved, with its biological activity level increased by 10–30 times (17, 19, 20). Although inflammatory proteases are able to convert IL-33 into a more active mature form, they may also result in the inactivation of IL-33 through protein degradation. This degradation has been observed in chymotrypsin. In addition, the endogenous caspase-3 can cleave at the DGVDG site in the C-terminal IL-1-like domain of IL-33 to inactivate IL-33. This structure is specific to IL-33, indicating that IL-33 is strictly regulated in the process of apoptosis (21). There was evidence showing that recombinant caspase-3 and caspase-7 could cleave IL-33 *in vitro*. Caspase-1 had no direct effect on IL-33 but could inactivate IL-33 by activating caspase-7 (22). In addition, when IL-33 was released into the extracellular microenvironment, it was quickly inactivated by the formation of two disulfide bonds. The oxidation of cysteine residues resulted in conformational changes and subsequent reduction in binding affinity to ST2. This regulation mechanism occurred much faster than protein degradation (23). Therefore, after a 2-h exposure to allergens, no biologically active IL-33 could be detected in the alveolar lavage fluid (23). Furthermore, IL-33 was found to accumulate in several models within a few hours after release and was not detectable after 6 h (24–26). These also reflect that IL-33 is a short-acting protein, and its biological role *in vivo* is precisely regulated.

In both physical and pathological inflammatory conditions, the main cellular sources of IL-33 are not CD45+ hematopoietic cells. Endothelial cells, epithelial cells, fibroblasts, and myofibroblasts in humans and mice were demonstrated to be the main cells expressing IL-33 (27). In addition to the epithelial barrier tissue and lymphatic organs, IL-33 was abundantly expressed in the brain and eyes of mice and weakly expressed in visceral smooth muscle cells of the human gastrointestinal tract and urogenital tract. Although several studies suggested that CD45+ hematopoietic cells may be a source of IL-33 even a major source, stronger evidence is needed (28–34). In an IL-33 luciferin reporter mouse model, no IL-33 was detected in CD45+ hematopoietic cells (including macrophages, dendritic cells, T cells, B cells, eosinophil, and neutrophil) in the lung of the mice with allergic pneumonia (35). It cannot be ruled out that certain leukocyte subsets may produce low levels of functional IL-33, but more experiments are still needed to verify it.

### IL-33 Signaling Pathway

**Figure 1:**
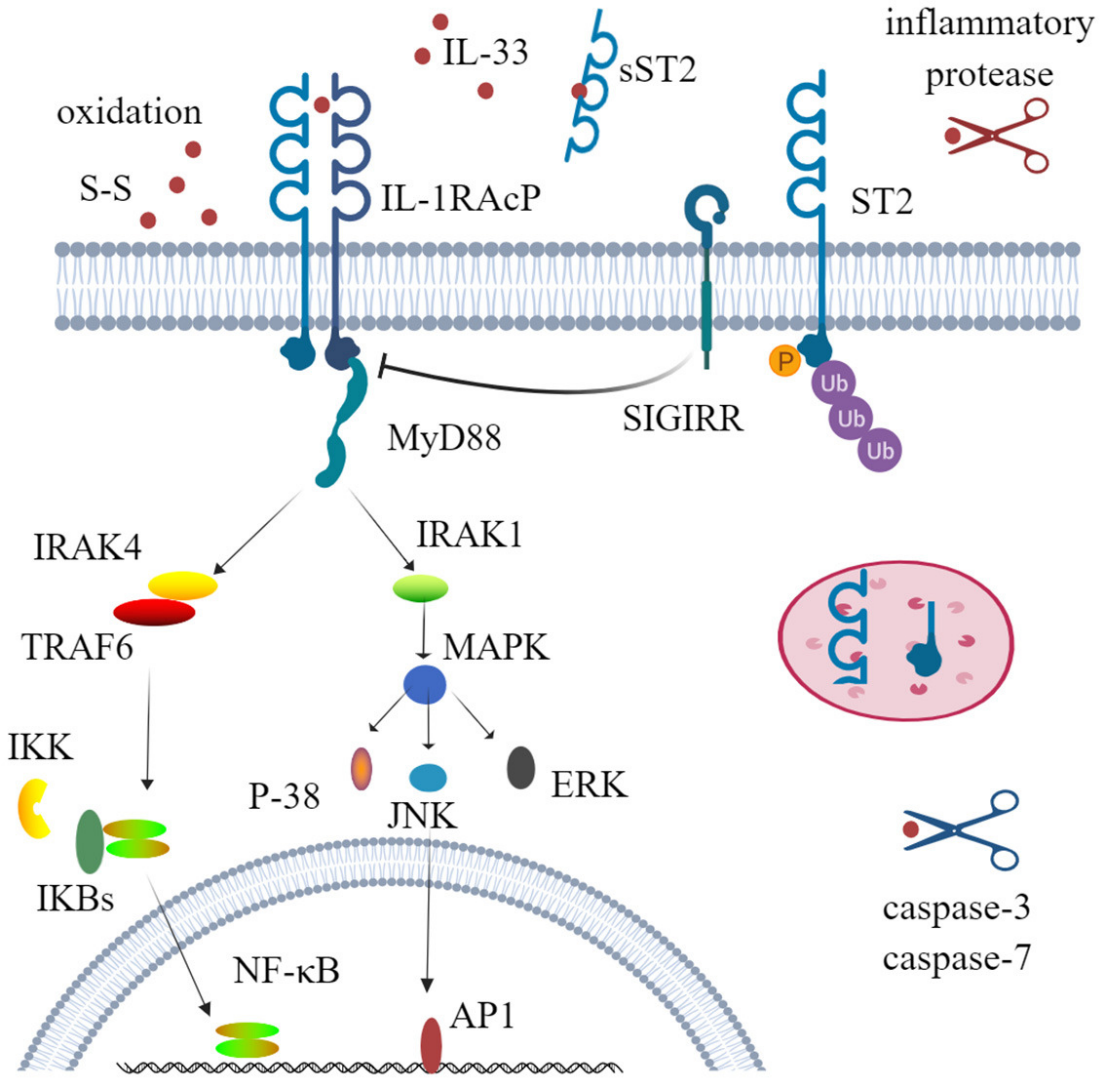
Physiological function and activity regulation of interleukin-33 (IL-33). IL-33 firstly binds to its transmembrane receptor serum stimulation-2 (ST2) to induce conformational changes; then ST2 interacts with IL-1RAcP and recruits the downstream adaptor MyD88, IRAK1, IRAK4, and TRAF6 via Toll/IL-1 receptor domains; and ultimately activates nuclear factor-kappa B (NF-κB) and mitogen-activated protein kinases (ERK, p38, and JNK). This signal pathway can also be inhibited by phosphorylation and ubiquitylation of ST2 and SIGIRR, which disrupt ST2 and IL-1RAcP dimerization. Several mechanisms are involved to regulate the activity of IL-33. Processing by inflammatory proteases can greatly increase (up to 30-fold) cytokine activity, while caspase-3 and caspase-7 lead to cytokine inactivity. Decoy receptor soluble ST2 (sST2) and rapid oxidation of IL-33 are also crucial mechanisms to limit the activity of IL-33. SIGIRR, single immunoglobulin domain IL-1 receptor-related molecule; IRAK, IL-1R-associated kinase; TRAF6, TNF receptor-associated factor 6; ERK, extracellular signal-regulated kinase.

The receptor ST2, also known as DER4, Fit-1, or T1, is one of the co-receptors of IL-33 and is mainly encoded by the IL-1RL1 gene. Before the discovery of IL-33, ST2 was considered as an orphan receptor, and now IL-33 is still the only ligand of ST2 (36, 37). Three isoforms of ST2 have been identified in humans, all of which are produced by alternative splicing: transmembrane receptor type (ST2L), soluble form (sST2), and variant ST2 (ST2V) (38–40). The soluble form acts as a decoy receptor to antagonize IL-33. When IL-33 binds to the transmembrane ST2 receptor, the membrane-anchored ST2 will recruit IL-1 receptor accessory protein (IL-1RAcP) to form a dimer, resulting in the dimerization of its intracellular domain. The adaptor protein myeloid differentiation protein 88 (MyD88) is recruited through the dimerization of Toll/IL-1 receptor (TIR) to activate downstream kinases. IL-1R-associated kinase 1 (IRAK1) and IL-1R-associated kinase 1 (IRAK4) and TNF receptor-associated factor 6 (TRAF6) are then activated, which ultimately leads to the activation of mitogen-activated protein kinases (MAPKs) and NF-κB transcription factor (41–44). This signal pathway is very similar to that of IL-1β and IL-18. Unlike IL-1RAcP, IL-33 stimulation induces a binding of ST2 with single immunoglobulin domain IL-1R-related molecule (SIGIRR) to form a complex, which can inhibit the formation of intracellular dimers and activate the ubiquitin–proteasome system (45) (Figure 1).

### Effects of IL-33 on Tissue Cells and Innate Immune Cells

**Figure 2:**
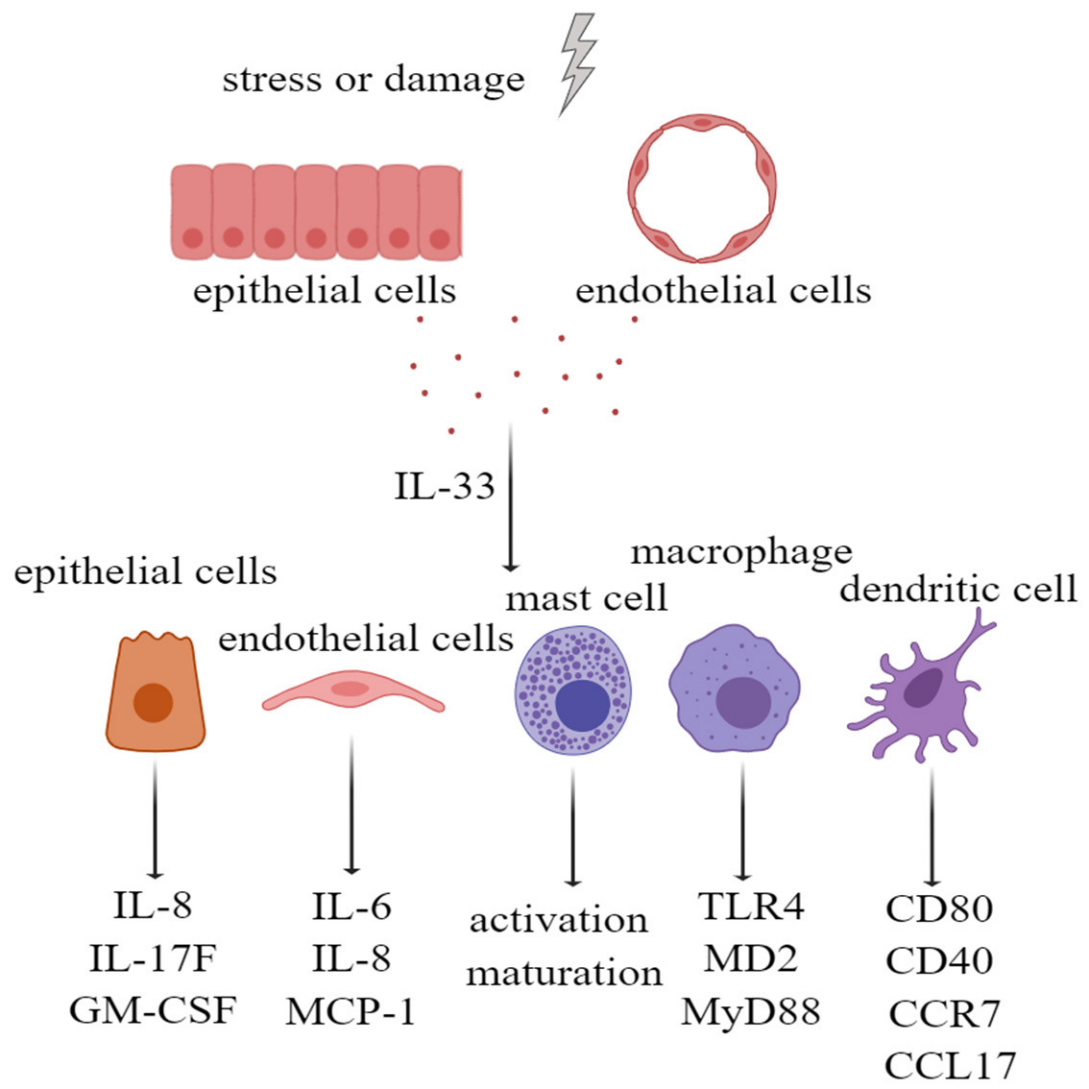
Interleukin-33 (IL-33), as alarmin, activates tissue cells and a variety of innate immune cells. Extracellular IL-33, acting on bronchial epithelial cells, led to upregulation of IL-8, IL-17F, and GM-CSF. IL-33 also upregulated IL-6, IL-8, and MCP-1 by stimulating pulmonary endothelial cells. IL-33 not only stimulated the activation and maturation of mast cells, and upregulation of TLR4, MD2, and MyD88 in macrophages, but also upregulated CD80, CD40, CCR7, and CCL17 in dendritic cells. GM-CSF, granulocyte-macrophage colony stimulating factor; MCP-1, monocyte chemoattractant protein-1; TLR-4, Toll-like receptor 4; MD2, myeloid differentiation protein-2; MyD88, myeloid differential protein-88; CCR7, cxc chemokine receptor 7; CCL17, chemokine (C-C motif) ligand 17.

Many types of cells express IL-33 receptors, including epithelial cells, endothelial cells, fibroblasts, and osteoblasts. IL-33 activates extracellular signal-regulated kinase (ERK) and p38 MAPK signaling pathways in primary human lung epithelial and endothelial cells to produce IL-8, which are associated with chronic airway inflammation (46). In addition, the IL-33 receptor ST2 is expressed on a variety of innate immune cells. IL-33/ST2 activation in mast cells not only promotes the activation and maturation of mast cells but also enhances Th17 response during airway inflammation (47, 48). In macrophages, IL-33/ST2 signaling enhances their activation by upregulating Toll-like receptor 4 (TLR4), myeloid differentiation protein-2 (MD2), and MyD88 (49). In the dendritic cells, the administration of IL-33 not only increases the levels of CD80, CD40, and C-C motif chemokine receptor 7 (CCR7) but also increases the production of IL-5, C-C motif chemokine ligand 17 (CCL17), and tumor necrosis factor alpha (TNF-α) (50). Therefore, IL-33, as an alarmin and damage-associated molecular pattern (DAMP) molecule, can activate tissue cells and innate immune cells; upregulate costimulatory molecules, adhesion molecules, and chemokines; and initiate and maintain innate immunity (Figure 2).

### IL-33 Indirectly Promotes the Production of IFN-γ

**Figure 3:**
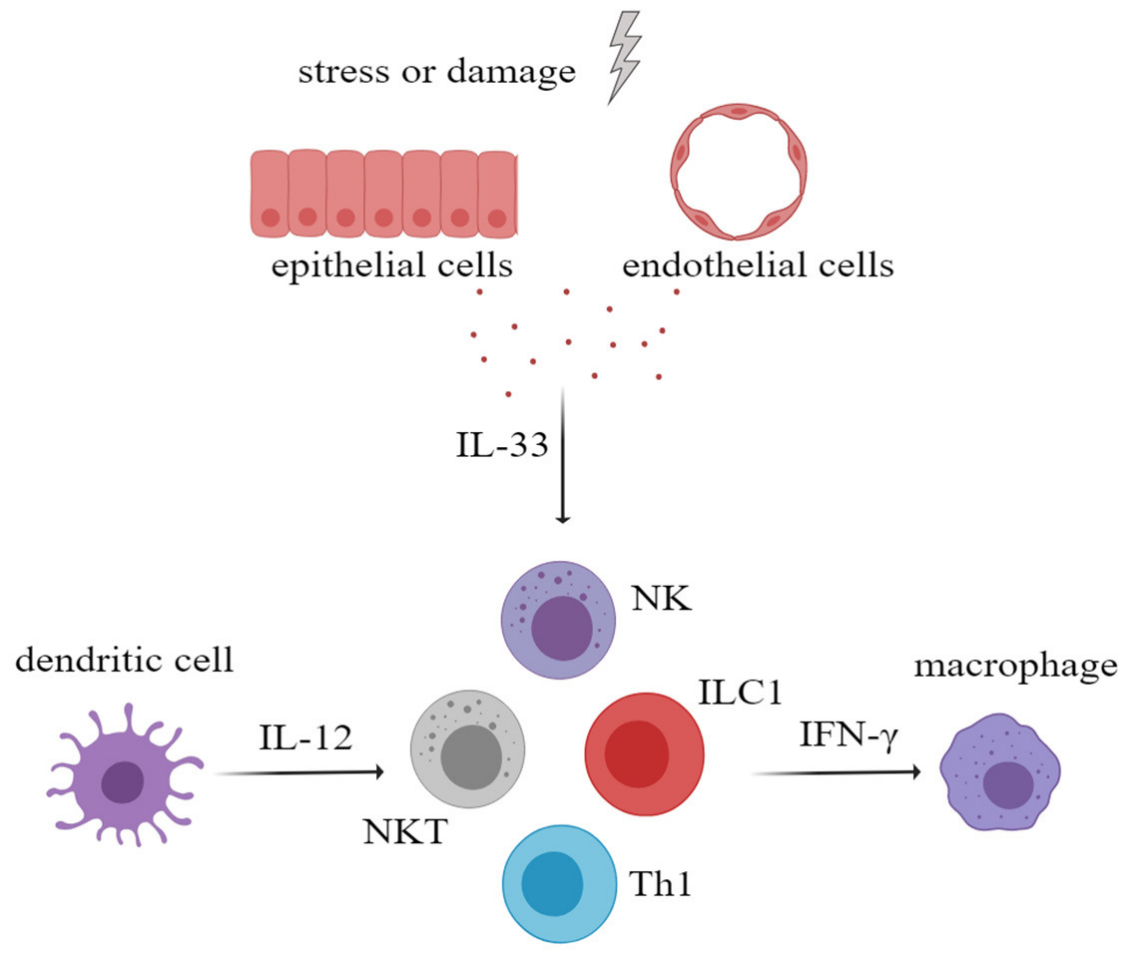
After activation of innate immune cells, especially myeloid dendritic cells, large amounts of interleukin-12 (IL-12) were secreted. IL-33 and IL-12 had a synergistic effect in stimulating NK cells, NKT cells, ILC1, and Th1 to secrete more IFN-γ, which will aggravate tissue damage. NK cells, natural killer cells; NKT cell, natural killer T cell; ILC, innate lymphoid cell.

Interferon-gamma (IFN-γ) plays an important role in the development of rheumatic immune disease. IFN-γ can activate macrophages or other immune cells to aggravate tissue damage and can promote the ectopic expression of MHC class II antigens on tissue cells, which may contribute to the presentation of autoantigen. Several studies have proven that IL-33, in the presence of IL-12, can increase the secretion of IFN-γ by NK cells, natural killer T cells (NKT cells), ILC1 cells, and Th1 cells (51–54). Therefore, in the progression of rheumatic disease, IL-33 may increase the production of IFN-γ and may amplify the immune effects (Figure 3). In addition, IL-33 is also thought to increase antibody levels in the immune response.

### IL-33 Promotes Tissue Repair and Fibrosis

**Figure 4:**
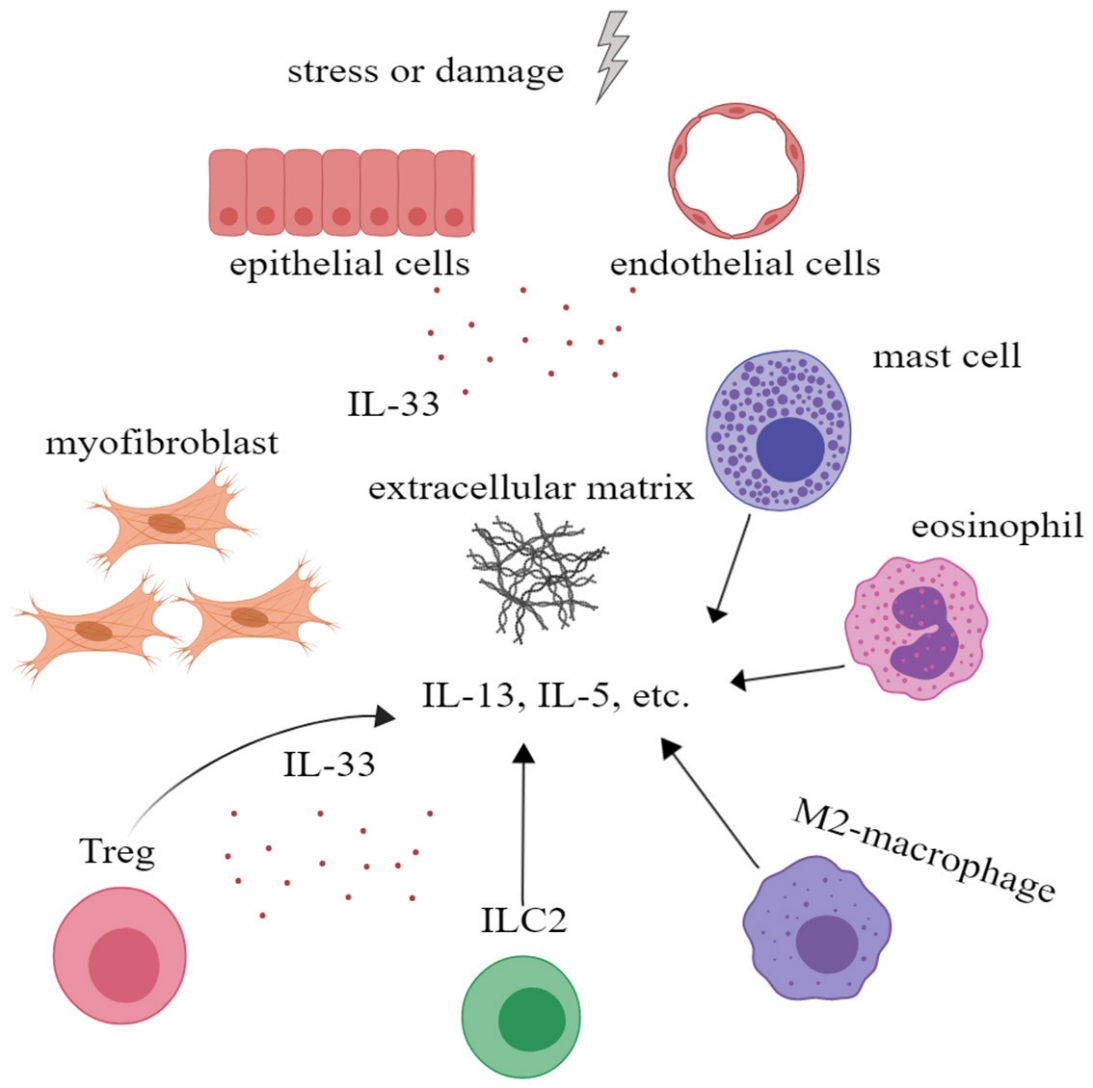
Interleukin-33 (IL-33) could promote tissue repair and fibrosis. IL-33 could promote the proliferation of Treg to further alleviate inflammation. IL-33 could also promote extracellular matrix deposition and tissue fibrosis by acting on mast cells, eosinophil, M2 macrophage, ILC2 cells, and Treg to increase the secretion of IL-5, IL-13, and other cytokines. ILC, innate lymphoid cell; Treg, regulatory T cell.

Although IL-33 is an alarmin and is involved in inflammatory processes, there is evidence that IL-33 plays a role in wound healing and fibrosis. IL-33/ST2 signal enables M2 macrophages to promote the closure of damaged epidermis and angiogenesis, etc. In addition, M2 macrophages are also involved in tissue remodeling and fibrosis (55). In addition, IL-33 can act on eosinophils, mast cells, and ILC2 to increase their production of IL-13 and IL-5, which are closely related to fibrosis (56). More importantly, IL-33 can increase the number of ST2+ regulatory T cell (Treg), a pivotal type of cells in fibrogenesis and immunosuppression (57). IL-33 is also believed to affect the activity of matrix metalloproteinases and promote the deposition of extracellular matrix (56). All these indicate that IL-33 plays an important role in tissue repair and fibrosis (Figure 4).

## IL-33/ST2 Axis in Rheumatic Diseases

**Figure 5:**
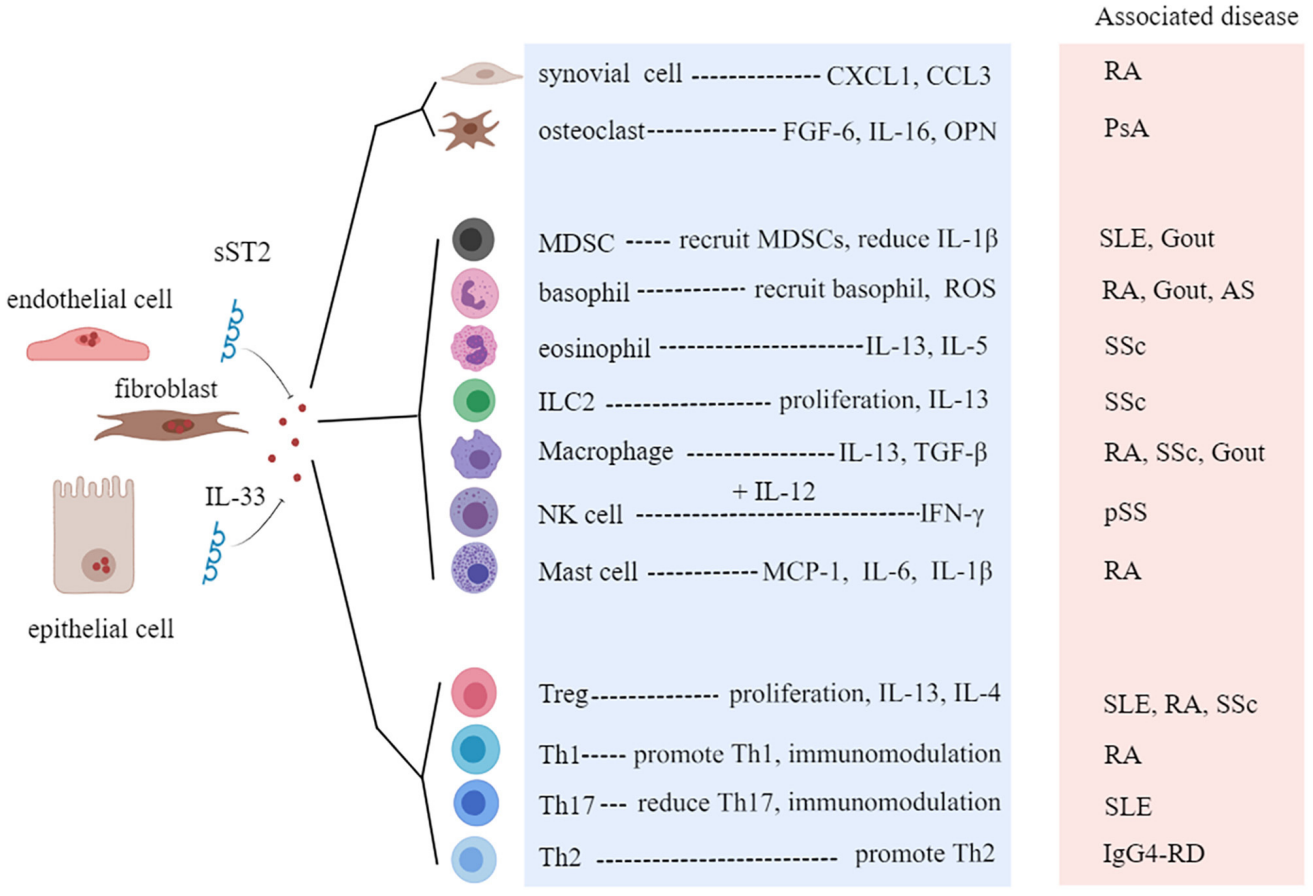
Roles of interleukin-33 (IL-33) in rheumatic diseases. sST2, soluble ST2; CXCL1, C-X-C motif chemokine ligand 1; FGF-6, fibroblast growth factor 6; OPN, osteopontin; MDSC, myeloid-derived suppressor cell; ROS, reactive oxygen species; ILC, innate lymphoid cell; Treg, regulatory T cell; RA, rheumatoid arthritis; SLE, systemic lupus erythematosus; pSS, primary Sjögren's syndrome; SSc, systemic sclerosis; PsA, psoriatic arthritis; IgG4-RD, IgG4-related disease; AS, ankylosing spondylitis.

Rheumatic diseases are immune-mediated chronic inflammatory syndromes, which are characterized by the hyperactivity of effector Th1 cells and Th17 cells, dysfunction of Tregs, activation of autoreactive B cells, and production of autoantibodies (58). A growing number of studies have found that the level of IL-33 is associated with the severity of rheumatic disease, indicating that IL-33 and ST2 may be potential targets for predicting the development of disease and improving the clinical outcomes (59, 60). Next, we will discuss the role of the IL-33/ST2 axis in several common rheumatic diseases (Table 1, Figure 5).

**Table 1:** Expression and mechanisms of IL-33/ST2 in rheumatic diseases.

**Disease**	**Role of IL-33/ST2 in disease pathogenesis**	**Referencess**
SLE	IL-33 and sST2 levels were increased in the serum of SLE patients. IL-33 neutralization had a protective effect in MRL/lpr mice, which was associated with the increase of regulatory T cells and myeloid-derived suppressor cells and the reduction of Th17 cells and pro-inflammatory factors. IL-33 might be involved in innate lymphoid cell disorder in the peripheral blood of SLE patients.	(61–64)
RA	IL-33 levels were related to the severity and activity of RA. IL-33 enhanced TNF-α-dependent effects in synovial fibroblasts. ST2^−/−^ and ST2 neutralization in the CIA model alleviated arthritis symptoms, while administration of IL-33 exacerbated. IL-33 stimulates mast cells to produce pro-inflammatory factors. IL-33 stimulated macrophages and synovial cells to produce chemokines, which recruited neutrophils.	(65–71)
pSS	IL-33 and sST2 levels were increased in the serum of pSS patients. IL-33 promoted the release of IFN-γ in NK and NKT cells when combined with IL-12 and/or IL-23.	(72–74)
SSc	IL-33 and sST2 levels were increased in the serum of SSc patients. IL-33 levels were correlated with skin lesions, degree of sclerosis. IL-33 polarized M2 macrophages to produce TGF-β1 and IL-13, induced ILC2 proliferation, increased eosinophils and the level of IL-13, and induced Treg dysfunction.	(75–79)
PsA	IL-33 levels were increased in the serum of patients. IL-33 increased the gene expression of the pro-osteoclastogenic factor associated with bone damage.	(80, 81)
Gout	IL-33 levels were increased in joint synovial fluid. Exogenous administration of IL-33 aggravated the production of ROS and recruitment of neutrophils, while knocking out ST2 alleviated the oxidative stress and neutrophils recruitment. IL-33 stimulated macrophages to produce CXCL1, CCL 3, and IL-1β. IL-33 recruited bone marrow-derived suppressor cells and reduced the production of IL-1β.	(82–84)
IgG4-RD	IL-33 levels were increased in the serum of patients. Prednisolone treatment decreased the serum concentration of IL-33. IL-33 activated the Th2 immune response and promoted tissue fibrosis.	(85–87)
AS	Serum IL-33 levels were elevated in the patients with AS. IL-33 enhanced the production of TNF-α and IL-6 in peripheral blood mononuclear cells and induced neutrophil migration. IL-33 was used as a predictor of the therapeutic effect of infliximab in the treatment of AS.	(88–91)
IIM	Serum sST2 levels were elevated and correlated with CRP, CK, and LDH.	(92, 93)
AOSD	Serum IL-33 and sST2 levels were elevated and correlated with ferritin levels.	(94)
BD	Serum and skin tissue of IL-33 and sST2 levels were elevated in patients.	(95, 96)

*SLE, systemic lupus erythematosus; RA, rheumatoid arthritis; CIA, collagen-induced arthritis; pSS, primary Sjögren's syndrome; NK, natural killer cells; NKT, natural killer T cells; SSc, systemic sclerosis; PsA, psoriatic arthritis; ROS, reactive oxygen species; IgG4-RD, IgG4-related disease; AS, ankylosing spondylitis; IIM, idiopathic inflammatory myopathies; CRP, C-reactive protein; CK, creatine kinase; LDH, lactate dehydrogenase; AOSD, adult-onset Still's disease; BD, Behcet's disease*.

### Systemic Lupus Erythematosus

SLE is a chronic connective tissue disease of unknown etiology with multiple systemic involvements. The male-to-female ratio is about 1:9, and it is a major cause of death in young women with chronic inflammatory diseases (97). The prevalence of SLE is (30.13–70.41)/100,000 in China. The level of serum IL-33 was reported to be significantly higher in patients with SLE than that in healthy subjects and was positively correlated with erythrocyte sedimentation rate (ESR), C-reactive protein (CRP), IgA, and Sjögren's syndrome antigen B (SSB) antibody levels (61). On the other hand, the serum soluble ST2 (sST2) level in patients was also significantly increased and was positively correlated with the level of anti-double-stranded DNA (dsDNA) antibodies and the disease activity index, while it was negatively correlated with the complement C3 (62). In addition, Guo et al. suggested that IL-33 and other pro-inflammatory cytokines might be involved in innate lymphoid cell disorder with a higher frequency of ILC1 in the peripheral blood of SLE patients (63). However, there were also other studies showing reduced or similar levels of serum IL-33 in SLE patients compared with healthy controls (62). This discrepancy is probably due to the detection efficacy of the enzyme-linked immunosorbent assay (ELISA) kit or the multiple roles of IL-33 plays in the disease (98). To further explore its effect, Li et al. found that the administration of neutralizing antibodies against IL-33 could reduce the mortality, serum anti-dsDNA level, and immune complex deposition in MRL/lpr lupus mice. The protective effect was associated with the increase of regulatory T cells and myeloid-derived suppressor cells and the reduction of Th17 cells and pro-inflammatory factors (64). These studies also indirectly supported the finding that two polymorphisms of the IL-33 gene (rs1929992-G and rs1891385-C) were associated with increased susceptibility to SLE (99–101). However, further studies are required to further explore the corresponding mechanism.

### Rheumatoid Arthritis

RA is an autoimmune disease with erosive arthritis as the main manifestation and synovitis as the pathological basis (102). The male-to-female ratio is 1:4. In severe cases, patients with uncontrolled active RA may develop joint deformities and disabilities. The prevalence of RA in China is 0.28–0.41%. Immunopathology showed that the differentiation of Th1 lymphocytes increased and that the number of Treg decreased in RA patients (103). Elevated IL-33 levels were found in both serum and local joint synovial fluid in patients with RA (65, 66, 103). Serum sST2 levels were also elevated (68). Moreover, IL-33 levels were associated with RA severity parameters, such as rheumatoid factor (RF), anti-cyclic citrullinated peptide (anti-CCP) antibodies, IL-6, ESR, lung involvement, and bone erosion (68, 104–106). IL-33 was also expressed in synovial fibroblasts (69). Inflammatory factors (such as TNF-α) could stimulate synovial fibroblasts to produce IL-33; and IL-33 not only upregulated matrix metalloproteinase-3 (MMP-3), IL-8, and IL-6 but also upregulated B-cell lymphoma-2 (Bcl-2) to inhibit apoptosis and promote proliferation (107). Furthermore, the level of serum IL-33 also had a certain significance for the prediction of patient's response to the biological agents. For example, in patients with poor response to tumor necrosis factor inhibitor (TNFi), serum and synovial fluid IL-33 levels continued to rise (70). In the mouse model, IL-33 mRNA levels increased in the early stages of collagen-induced arthritis (CIA) (65). With the use of ST2 knockout mice and ST2 neutralizing antibodies in the CIA model, suppression of ST2 signaling alleviated arthritis symptoms and reduced levels of IL-17, TNF-α, and IFN-γ (65, 69). In contrast, the administration of IL-33 resulted in the exacerbation of arthritis (108). Interestingly, this effect was absent in mice lacking mast cells. Further studies showed that mast cells expressed high levels of ST2, which responded to IL-33 by producing various pro-inflammatory factors such as monocyte chemoattractant protein-1 (MCP-1), IL-6, and IL-1β (69). In addition, IL-33 could also recruit neutrophils by stimulating macrophages and synovial cells to release chemokines (such as CXCL1 and CCL3) (71). Although most studies support the deleterious effect of the IL-33/ST2 axis in the pathogenesis of RA, there are still some studies with opposite results. For example, repeated administration of IL-33 in the early and late stages of CIA mice models could relieve symptoms of arthritis. The protective mechanism might involve the regulation of immunity and the proliferation of Tregs (109). Despite the controversy, research from the gene polymorphism suggests that downregulating the expression of IL-33 shows resistance to disease. Further studies are needed to explore the specific role and molecular mechanisms of IL-33 in RA.

### Primary Sjögren's Syndrome

pSS is a chronic systemic rheumatic disease mainly involving salivary and lacrimal glands. The male-to-female ratio is 1:(9–20) (110). The prevalence of pSS in China is 0.28–0.41%. Multiple studies have described the pathogenic role of the IL-33/ST2 axis in patients with pSS (72, 73, 111–113). Compared with those in the control group, serum IL-33 and sST levels were elevated in pSS patients. Although serum IL-33 levels and EULAR Sjögren's syndrome disease activity index (ESSDAI) or lymphocyte infiltration were not correlated, serum sST2 levels were significantly correlated with ESSDAI, disease duration, and thrombocytopenia (72, 73). Immunohistochemical staining showed that IL-33 and its receptors (ST2 and IL-1RAcP) were expressed in the salivary glands. The expression of IL-33 in patients with pSS showed a dynamic pattern: IL-33 was significantly increased in salivary glands with Chisholm scores of 2 and 3 but was expressed at a lower level in salivary glands with Chisholm scores of 1 and 4. Its receptors (ST2 and IL-1RAcP) were expressed in a similar pattern (73). In addition, a recent study showed that the levels of IL-33 in the tears of patients with pSS were also significantly increased. Furthermore, IL-33 levels were correlated with the degree of ocular involvement and levels of IL-4 and IL-5 in tears (74). However, IL-33 alone did not lead to the release of pro-inflammatory factors. But when combined with IL-12 and/or IL-23, it promoted the release of IFN-γ by up to 10 times in NK and NKT cells. Moreover, TNF-α, IL-1β, and IFN-γ in the inflammatory environment could further increase the activation of IL-33, forming positive feedback (60). Therefore, the targeting IL-33/ST2 axis may be a promising treatment option for pSS.

### Systemic Sclerosis

SSc is a rheumatic disease with unknown etiology characterized by the deposition of the extracellular matrix and diffuse skin thickening (114). The male-to-female ratio is 1:(3–7), and the lence rate in China is about 0.026%. It was reported that the serum IL-33 level in patients with early SSc was higher than that of healthy controls and patients with advanced SSc. This might be due to the activation of endothelial cells in the early stages of the disease, and elevated IL-33 level was positively correlated with skin lesions, degree of sclerosis, and degree of pulmonary fibrosis (75, 76). In skin biopsies of healthy subjects, ST2 was only expressed in fibroblasts and endothelial cells at a low level, while IL-33 was constitutively expressed in keratinocytes and endothelial cells (77). However, in the early stage of SSc, ST2 was highly expressed in endothelial cells, macrophages, T cells, B cells, and myofibroblasts of the affected organs, while the expression of IL-33 in tissues was not significantly increased until the late stage of SSc (77). In fact, the IL-33/ST2 axis could polarize M2 macrophages to promote the production of TGF-β1 and IL-13 and could also induce the proliferation of ILC2 to promote the accumulation of eosinophil granulocyte and expression of IL-13. Moreover, *in vitro* experiments have shown that IL-33 could induce the differentiation of Tregs into Th2-like cells, resulting in the production of IL-4 and IL-13 and local Treg dysfunction (77–79). The study in our laboratory also found that IL-33 could directly promote the proliferation of primary human skin fibroblasts and their expression of collagen. The administration of ST2 neutralizing antibody was able to effectively alleviate bleomycin-induced skin fibrosis in mice. Moreover, the polymorphism of IL-33 gene rs7044343 is associated with SSc-associated dyspnea in the Chinese population and SSc susceptibility in the Turkish population (115, 116). However, further research is needed to determine the therapeutic effect of IL-33/ST2 targeting therapy in human SSc.

### Psoriatic Arthritis

PsA is a chronic, inflammatory, musculoskeletal disease affecting the skin, peripheral joint, spine, nails, and entheses (117). It was reported that up to 30% of patients with psoriasis might develop PsA (118). Several studies showed that IL-33 not only played a role in the development of psoriasis but also participated in the progress of PsA (119). One study detected elevated serum IL-33 in patients with PsA, but there was no correlation with osteoclastogenesis-related cytokines and PSA joint activity index (PSAJAI) (80). In another study, however, IL-33 was not detected in serum and synovial fluid from PsA patients but only in endothelial cells of the synovium and synovial fibroblast (120). Another study focused on the effects of skin inflammation on bone damage. IL-33 together with IL-17 increased the gene expression of the pro-osteoclastogenic factor, such as fibroblast growth factor (FGF-6), IL-16, and osteopontin (OPN). Moreover, IL-33, together with OPN, IL-17, and TNF-α, could also induce the release of bone contributing factor receptor activator of NF-κB ligand (RANKL) in the skin, thus inducing the differentiation of osteoclast precursor (OCP) into monocytes (81). IL-33 was also expressed in the synovium in a mouse model of PsA. But there was no difference between IL-33^−/−^ and wild-type (WT) mice in frequencies of Treg, Th1, and Th17 cells in this model (121). In conclusion, the present studies suggest that IL-33 is involved in the development of human PsA, while studies in mouse models are limited. Further studies are needed to obtain more evidence.

### Gout

Gout is an inflammatory disease characterized by the deposition of uric acid crystals in the joints (122). The male-to-female ratio is 15:1, and its incidence in China is 1% to 3%. It was reported that higher levels of IL-33 and neutrophil counts were detected in joint synovial fluid in patients with gout than those in osteoarthritis (82). Direct injection of uric acid crystals into the articular cavity could induce acute attacks of gout in mice. In this mice model, exogenous administration of IL-33 could aggravate the production of reactive oxygen species, recruitment of neutrophils, and hyperalgesia. Correspondingly, knocking out ST2 could significantly alleviate oxidative stress and reduce neutrophils' recruitment into the ankle joint. It was also found that macrophages in gout could produce IL-33 and increase CXCL1, CCL 3, and IL-1β through an autocrine pattern. These results supported the pathogenic role of the IL-33/ST2 axis in gout (83). Paradoxically, IL-33 was also believed to alleviate the mouse peritonitis model induced by uric acid crystals and to reduce neutrophil counts as well as the production of IL-1β and IL-6 (84). The mechanism was associated with the recruitment of bone marrow-derived suppressor cells by IL-33, which reduced the production of IL-1β in the peritoneal cavity (84). These results also precisely reflect the distinct action patterns of IL-33 in different sites and under different pathological conditions.

### IgG4-Related Disease

The IgG4-related disease (IgG4-RD) is an idiopathic, fibroinflammatory disease characterized by elevated serum IgG4 levels, tumefaction, and tissue infiltration by IgG4-positive plasma cells (123). The ratio of male to female is ~(2–3):1. The current incidence in China is unknown. However, with the improvement in disease cognition and detection, the number of patients is gradually increasing. Furukawa et al. found that IL-33 could act as an inducer of Th2 response in IgG4-RD (85). Further studies found that in patients with IgG4-related autoimmune pancreatitis, plasmacytoid dendritic cells could produce IL-33 and interferon alpha (IFN-α), which were closely related to the fibrosis of the disease. The authors further validated these results in a mice model and demonstrated that depletion of plasmacytoid dendritic cells and blockade of signaling pathways related to type 1 interferon and IL-33 could prevent chronic fibrosis (124). Treatment with prednisolone was able to improve the swelling of the pancreas with a significant reduction of serum IFN-α and IL-33, but the serum IgG, IgG4, and IgE concentrations only slightly decreased. This suggested that the IFN-α/IL-33 axis may be a better biomarker reflecting the disease activity of IgG4-RD compared with serum levels of IgG, IgG4, and IgE (86, 125, 126). There is no correlation between serum IL-33 and serum IgG4 or IgG4:IgG ratio (126). Another study on IgG4-related sialadenitis demonstrated that TLR7-positive M2 macrophage was able to produce high levels of IL-33 *in vitro*, which activated the Th2 immune response and promoted tissue fibrosis in IgG4-RD (87). In conclusion, IL-33 plays an important role in the development of IgG4-related diseases as an important inducer of type 2 immunity and an important pro-fibrogenic factor. However, the specific mechanism is still unclear.

### Ankylosing Spondylitis

AS is a chronic, inflammatory rheumatic disease affecting the spine and sacroiliac joints. The main clinical features are inflammatory back pain, bone erosion, and syndesmophyte formation (127). In China, the prevalence of AS is 0.25~0.5%, and the ratio of male-to-female is about 4–1. Due to the lack of suitable animal models, all studies of IL-33 in AS have been conducted in humans. Serum IL-33 levels were reported to be elevated in patients with AS, especially in patients with active AS (88). In addition, IL-33 enhanced the production of TNF-α and IL-6 in peripheral blood mononuclear cells (PBMCs) and induced neutrophil migration when the dose of IL-33 exceeded 10 ng/ml (89). Another study explored the relationship between IL-33 gene polymorphisms and disease susceptibility and found that AS patients carrying the IL-33 rs16924159 AA genotype had higher disease activity and a worse response to anti-TNF therapy (90). But overall, IL-33 could be still used as a predictor of the therapeutic effect of infliximab in the treatment of AS (91). These results suggest that IL-33 is involved in the pathogenesis of AS and is a potential therapeutic target, but more studies are still needed.

### Other Rheumatic Diseases

IL-33 also played a role in other rheumatic diseases such as idiopathic inflammatory myopathies (IIM), adult-onset Still's disease (AOSD), and Behcet's disease (BD).

IIM is a chronic rheumatic disease, which can lead to skin and internal organ involvement. IIM includes dermatomyositis (DM) and polymyositis (PM). It was reported that serum sST2 levels were significantly elevated in DM and PM patients and decreased after treatment. In addition, serum sST2 levels were correlated with CRP, creatine kinase (CK), and lactate dehydrogenase (LDH) (92). In another study, serum IL-33 could not be detected in the majority of IIM patients, but serum sST2 levels were elevated and even much higher in patients with anti-signal recognition particle (anti-SRP) antibodies (93). Considering the abnormal expression of sST2 and the short detection time window of IL-33, it can be speculated that IL-33 may be involved in the pathogenesis of IIM.

AOSD is a rare systemic inflammatory disorder, which is characterized by high spiking fever, an evanescent rash, polyarthralgia, arthritis, and hepatosplenomegaly. It was reported that serum levels of IL-33 and sST2 were elevated in patients with active AOSD; and serum IL-33 levels correlated with systemic score, ESR, ferritin levels, and aspartate transaminase levels, while serum soluble ST2 levels correlated only with ferritin levels (94). These results indicated that the IL-33/ST2 signaling pathway may play a role in the pathogenesis of AOSD.

BD is a multisystem inflammatory disease, characterized by recurrent oral ulceration, skin lesions, genital ulcerations, and uveitis. Serum and skin tissue of IL-33 and sST2 levels were reported to be elevated in patients with BD, and sST2 is associated with ESR and CRP (95). Another study reported that in BD patients of Iran, the expression of IL-33 mRNA in PBMCs was much higher than in healthy controls, and rs1342326 T/G polymorphism of the IL-33 gene might contribute to the genetic susceptibility to BD (96). These results suggest that IL-33 may play an important role in the pathogenesis of BD.

## Discussion

In this review, we summarize the role of the IL-33/ST2 axis in rheumatic diseases by summarizing the evidence from clinical patients, mouse models, and *in vitro* cell culture. IL-33 is characterized as an alarmin, with ILC2, Th2, and Tregs as the main target cells in immune system. Because of the complexity and functional diversity of IL-33, it may play distinct roles in different stages of disease and different immune microenvironments. For example, administration of exogenous IL-33 with different treatment duration, different concentrations, or different stage of disease may result in different, even opposite, therapeutic effects. Nevertheless, previous studies have demonstrated a detrimental role of IL-33/ST2 axis in RA, scleroderma, SLE, psoriasis, and gout. The potential mechanisms may involve the immunomodulation, fibrogenesis, and tissue repair.

IL-33 is able to promote the polarization of macrophages, activate mast cells ILC2, and induce eosinophil activation. We and others have shown that some tissue cells including epithelial cells and fibroblasts also express IL-33 and its receptor ST2. IL-33 can also participate in the pathogenesis of rheumatic disease by interacting with ST2-expressing tissue cells, such as cardiomyocytes, oligodendrocytes, epithelial cells, and endothelial cells. The activation, dysfunction, and destruction of these cells are directly involved in the development of many rheumatic diseases. In general, IL-33/ST2 axis plays a detrimental role in both early and advanced stages of most rheumatic diseases. In the early stages of the disease, IL-33 can be released from damaged epithelial cells acting as an alarmin to activate other local tissue cells and recruit immune cells. In addition, IL-33 seems to interact with various cytokines such as IL-12 to produce more IFN-γ in the early inflammation environment. In contrast, during the advanced repair and fibrosis stages, IL-33 as an important pro-fibrogenic factor may play a protective role in maintaining the integrity of the barrier system. These characteristics determine the pleiotropy and complexity of IL-33.

## Conclusion

In summary, the sources and targets of IL-33 involve a variety of cell types. Although great advances have been made in recent years, more evidence is needed to clarify the exact role of the IL-33/ST2 axis in rheumatic diseases.

## Author Contributions

All authors listed have made a substantial, direct and intellectual contribution to the work, and approved it for publication.

## Funding

This work was supported by grants from the National Natural Scientific Foundation of China (No. 81771754) and Tongji Hospital Clinical Research Flagship Program (No. 2019CR206).

## Conflict of Interest

The authors declare that the research was conducted in the absence of any commercial or financial relationships that could be construed as a potential conflict of interest.

## Publisher's Note

All claims expressed in this article are solely those of the authors and do not necessarily represent those of their affiliated organizations, or those of the publisher, the editors and the reviewers. Any product that may be evaluated in this article, or claim that may be made by its manufacturer, is not guaranteed or endorsed by the publisher.
